# Etymologia: *Campylobacter*

**DOI:** 10.3201/eid1908.ET-1908

**Published:** 2013-08

**Keywords:** etymologia, *Campylobacter*, bacteria, vibrio-like, curved rod, enteric infections, foodborne infections, diarrhea, gastroenteritis

## *Campylobacter* [kam′′pə-lo-bak′tər]

From the Greek *kampylos* (curved) and *baktron* (rod), a genus of gram-negative curved or spiral rods that is among the most common causes of foodborne diarrheal illness worldwide. Illness caused by *Campylobacter* spp. was first described by Theodor Escherich in 1886, but they were not successfully isolated from human fecal samples until 1972. For many years, they were classified among the vibrios, but Sebald and Véron proposed the genus *Campylobacter* in 1963 for these “slender, curved bacilli” that differ from the classical cholera and halophilic vibrios.
